# tRNA^Glu^-derived fragments from embryonic extracellular vesicles modulate bovine embryo hatching

**DOI:** 10.1186/s40104-024-00997-7

**Published:** 2024-03-01

**Authors:** Yuan Fan, Krishna Chaitanya Pavani, Katrien Smits, Ann Van Soom, Luc Peelman

**Affiliations:** 1 Department of Veterinary and Biosciences, Faculty of Veterinary Medicine, Ghent University, Heidestraat 19, 9820 Merelbeke, Belgium; 2 Department of Internal Medicine, Reproduction and Population Medicine, Faculty of Veterinary Medicine, Ghent University, Salisburylaan 133, 9820 Merelbeke, Belgium; 3 Department for Reproductive Medicine, Ghent University Hospital, Corneel Heymanslaan 10, 9000 Ghent, Belgium

**Keywords:** Embryo, Extracellular vesicles, Hatching, tRNA fragments, tsRNAs

## Abstract

Transfer RNA-derived small RNAs (tsRNAs) have been shown to be involved in early embryo development and repression of endogenous retroelements in embryos and stem cells. However, it is unknown whether tsRNAs also regulate embryo hatching. In this study, we mined the sequencing data of a previous experiment in which we demonstrated that the microRNA (miRNA) cargo of preimplantation embryonic extracellular vesicles (EVs) influences embryo development. We thus profiled the tsRNA cargo of EVs secreted by blastocysts and non-blastocysts. The majority of tsRNAs was identified as tRNA halves originating from the 5´ ends of tRNAs. Among the 148 differentially expressed tsRNAs, the 19 nt tRNA fragment (tRF) tDR-14:32-Glu-CTC-1 was found to be significantly up-regulated in EVs derived from non-blastocysts. RT-qPCR assays confirmed its significant up-regulation in non-blastocyst embryos and their conditioned medium compared to the blastocyst group (*P* < 0.05). Inhibition of tDR-14:32-Glu-CTC-1 by supplementing antagomirs to the conditioned medium improved embryo hatching (*P* < 0.05). Transcriptomic analysis of embryos treated with tDR-14:32-Glu-CTC-1 antagomirs further showed differential expression of genes that are associated with embryo hatching and implantation. In summary, tDR-14:32-Glu-CTC-1 is up-regulated in non-blastocyst embryos and their secretions, and inhibition of tDR-14:32-Glu-CTC-1 promotes embryo hatching, while influencing embryo implantation-related genes and pathways. These results indicate that embryonic EVs containing specific tRFs may regulate preimplantation embryo development.

**Supplementary Information:**

The online version contains supplementary material available at 10.1186/s40104-024-00997-7.

## Background

Mammalian preimplantation embryo development is an orchestrated biological event encompassing fertilization, first cleavage and continuing cell divisions, compaction and lineage differentiation for blastocyst formation. At the blastocyst stage, embryos will hatch from the zona pellucida with the acquisition of competency for successful implantation [1]. However, implantation does not always succeed especially for embryos produced in vitro. It’s noteworthy that embryo implantation failure accounts for over 50% of pregnancy losses in both humans and cattle after in vitro production [1, 2]. Thus, the exploration of underlying mechanisms and signaling molecules related to embryo hatching and implantation would significantly enhance our understanding of embryo development and contribute to the improvement of assisted reproductive technology. In previous work, we highlighted the roles of specific embryonic miRNAs (miR-10b, miR-30c and miR-378a) in embryo development and quality [3–5]. Additionally, the content of EVs derived from both blastocyst and non-blastocyst conditioned media suggested a potential regulating role for other small non-coding RNAs (sncRNAs) besides miRNAs [6]. Therefore, identification and functional analysis of these novel sncRNA molecules will lead to a better knowledge of the molecular mechanisms involved in early embryo development.

tsRNAs, a lesser known kind of sncRNAs which were long considered as degenerate “junk RNA” of tRNAs, are cleaved at different sites from tRNAs [7, 8]. Based on the cleavage sites and length, tsRNAs can be categorized into two main types: tRNA halves (tiRNAs, around 30–34 nt) and tRFs (around 20 nt) [9]. To date, certain tiRNAs and tRFs have been identified as regulators of disease and other various biological processes, including cancers [10], liver injury [11] and skeletal muscle regeneration [12]. Several studies have suggested a regulatory role of tiRNAs in early embryogenesis. For instance, inhibition of tRNA^Gln-TTG^ derived small RNAs present in mature spermatozoa led a decrease of the first cleavage of porcine embryos and subsequently impaired embryo development [13]. Similar observations were made concerning tiRNAs in human zygotes [14]. Additionally, using zebrafish embryos as a model, the indispensable role of 5´ half of specific tRNAs in vertebrate early embryogenesis, primarily through promotion of transcription of their corresponding tRNA genes, was shown [15]. It is noteworthy, that all the prior studies were centered on tiRNAs, leaving the roles of tRFs in early embryo development unexplored. Hence, in this study, we re-analyzed the small RNA sequencing data on EVs derived from both bovine blastocyst and non-blastocyst conditioned media to uncover tRFs which may play a role in preimplantation embryo development.

## Materials and methods

### Small RNA-seq analysis

Small RNA-seq data were re-analyzed using SPORTS1.1 [16]. The raw data were first processed by TrimGlore to remove adaptors and were quality controlled. The obtained clean reads were sequentially mapped against the *Bos taurus* reference genome (ARS-UCD 1.2), miRBase v21.0 [17], rRNA database (collected from NCBI), GtRNAdb [18], piRbase v3.0 [19], Ensembl *Bos taurus* ncRNA (ARS-UCD1.2) [20] and Rfam v12.3 [21] by Bowtie [22]. After mapping, tsRNAs reads were characterized by SPORTS1.1 based on their original sites from tRNAs and tDRnamer v1.2 [23] was applied for tsRNAs naming, using the *Homo sapiens* (GRCh37/hg19) database as a reference because currently no bovine reference database is available. Differential expression analysis was performed with DESeq2 and only tsRNAs with both *P*_adj_ < 0.05 and |log_2_FC| > 1 were treated as differentially expressed tsRNAs.

### In vitro embryo production and sample collection

Routine in vitro bovine embryo production was performed under standard operating procedures in our laboratory as previously described [4, 5]. Briefly, bovine ovaries were obtained from Euro Meat Group (Mouscron, Belgium) and processed in 2 h. Once arrived, cumulus oocyte complexes from 4- to 8-mm diameter follicles were aspirated with a 18-gauge needle after washing ovaries three times with warm physiological saline containing 25 mg/mL kanamycin. Then, groups of 60 cumulus oocyte complexes were cultured in 500 μL TCM-199 (supplemented with 50 mg/mL gentamicin and 20 ng/mL epidermal growth factor) in 5% CO_2_ at 38.5 °C for maturation. After 22 h, a 45/90% Percoll gradient was used for frozen–thawed bovine spermatozoa separation. The final sperm concentration was adjusted to 1 × 10^6^ for fertilization. The matured cumulus oocyte complexes were put under gamete co-incubation in IVF-TALP medium (supplemented with 6 mg/mL BSA and 20 μg/mL heparin) for 21 h. After fertilization, presumed zygotes were selected for in vitro culture after vortexing. For group culture, each 25 presumed zygotes were placed in a 50 μL droplet of synthetic oviductal fluid (SOF) supplemented with ITS (5 μg/mL insulin + 5 μg/mL transferrin + 5 ng/mL selenium) and 4 mg/mL BSA. For individual culture, each presumed zygote was cultured in a 20 μL SOF droplet. Both droplets were covered with mineral oil at 38.5 °C in 5% CO_2_, 5% O_2_, and 90% N_2_. Embryo developmental rates were assessed based on the embryo morphology according to the IETS manual until 8 dpi (day post insemination) [24]. On 8 dpi, embryos were divided into non-blastocysts and blastocysts. For individual cultures, 12.5 μL conditioned medium from each droplet was collected for the following experiments. Four samples of individual conditioned medium were merged into a 50 μL sample as a biological replicate. Individual culture was performed only for conditioned media collected for tRFs expression validation.

### tsRNA agomir supplementing

Chemical synthesized tsRNA agomir, antagomir and their scrambled controls were all purchased from GenePharma (Shanghai, China). The sequences of oligonucleotides were as follows: tDR-14:32-Glu-CTC-1 agomir (5´-AGUGGUUAGGAUUCGGCGC-3´ and 3´-UUUCACCAAUCCUAAGCCG-5´), tDR-14:32-Glu-CTC-1 agomir scramble control (5´-UUCGUGAGCAGGUCGUGAG-3´ and 3´-UUAAGCACUCGUCCAGCAC-5´), tDR-14:32-Glu-CTC-1 antagomir (5´-GCGCCGAAUCCUAACCACU-3´) and tDR-14:32-Glu-CTC-1 antagomir scramble control (5´-UCCCAACAAGUUGCGCACC-3´). Oligonucleotides were supplemented into the conditioned medium of group cultured presumed zygotes with a final concentration of 1 μmol/L as previous described [3–5]. Three replicates for each group. The number of presumed zygotes are 288, 300 and 241 for control, agomir and agomir control group, respectively and the number of presumed zygotes are 275, 275 and 225 for control, antagomir and antagomir control group, respectively.

### Agomir intake assay

FAM-labeled tDR-14:32-Glu-CTC-1 agomir scramble control was purchased from GenePharma (Shanghai, China) and supplemented into the conditioned medium of group cultured presumed zygotes on 1 dpi. On 8 dpi, blastocysts were washed with 1 mg PVP/mL PBS for three times and then fixed in 4% paraformaldehyde under ambient temperature for 1 h. After fixing, blastocysts were stained with Hoechst 33342 (Thermo Fisher, Waltham, MA, USA) for 10 min and imaged by Leica DM5500B Fluorescence Microscope. Images were merged by ImageJ 8.0.

### RNA extraction

Before the RNA extraction of conditioned medium, 1 fmol of cel-miR-39 RNA (Norgen Biotek, ON, Canada) was added as spike-in reference. RNA extraction for both conditioned medium and embryos was performed using Qiagen miRNeasy Serum/Plasma Kit according to manufacturer’s instructions.

### RT-qPCR

Reverse transcription was performed using Mir-X™ miRNA First Strand Synthesis Kit (TaKaRa, CA, USA) according to the manufacturer’s protocol. A BioRad CFX 96 PCR detection system was used for qPCR. In brief, 5 μL TB Green (TaKaRa, Japan), 3.6 μL ddH_2_O, 1 μL cDNA template and 0.4 μL 10 nmol/L primer mix were mixed to form a 10 μL qPCR reaction solution. The PCR program used consisted of an initial template denaturation step before the actual PCR at 95 °C for 30 s, followed by 40 cycles of denaturation for 5 s at 95 °C, combined annealing-extension for 20 s at 60 °C. The reaction was ended by a melt curve step from 70 °C to 95 °C in 0.5 °C increments for 5 s. qPCR data were normalized using U6 (for embryos), cel-miR-39 and tDR-1:32-Gly-CCC-1 (for conditioned medium) as references with the 2^–∆∆Ct^ method. U6 and universal reverse primers were included in the RT kit and the sequences of the other primers were as follows: tDR-14:32-Glu-CTC-1 (5´-AGTGGTTAGGATTCGGCGC-3´), tDR-1:32-Gly-CCC-1 (5´-GCATTGGTGGTTCAGTGGTAGAATTCTCGCC-3´) and cel-miR-39 (5´-GCGCCGAATCCTAACCACT-3´). All primer efficiencies in this study were between 90% and 105%.

### Transcriptomics and bioinformatics

Control group and tDR-14:32-Glu-CTC-1 antagomir group were collected on 8 dpi for RNA-seq. For each replicate (*n* = 3), 20 embryos (10 hatched or hatching blastocysts and 10 not hatching blastocysts) were pooled. After RNA isolation, the RNA quality was examined using the Agilent 2100 Bioanalyzer system. 1 ng total RNA was applied for library construction followed tagmentation-based library construction protocol. Sequencing was performed on the BGISEQ-500 platform at BGI (Beijing, China). The *Bos taurus* reference genome (ARS-UCD 1.2) was chosen for genome mapping. The significance of the differentially expressed genes (DEGs) was defined by *P*_adj_ < 0.05. Kyoto Encyclopedia of Genes and Genomes (KEGG), Gene Ontology (GO) and gene set enrichment analysis (GSEA) were performed on the OmicShare online platform.

### Statistical analysis

All data are represented as the mean ± standard error (SE). Each experiment was repeated at least three times. The data were analyzed using a Student’s *t* test or one-way ANOVA followed by Tukey’s test using GraphPad prism 9 and the differences were considered statistically significant at *P* < 0.05 (*) and highly significant at *P* < 0.01 (**).

## Results

**Fig. 1:**
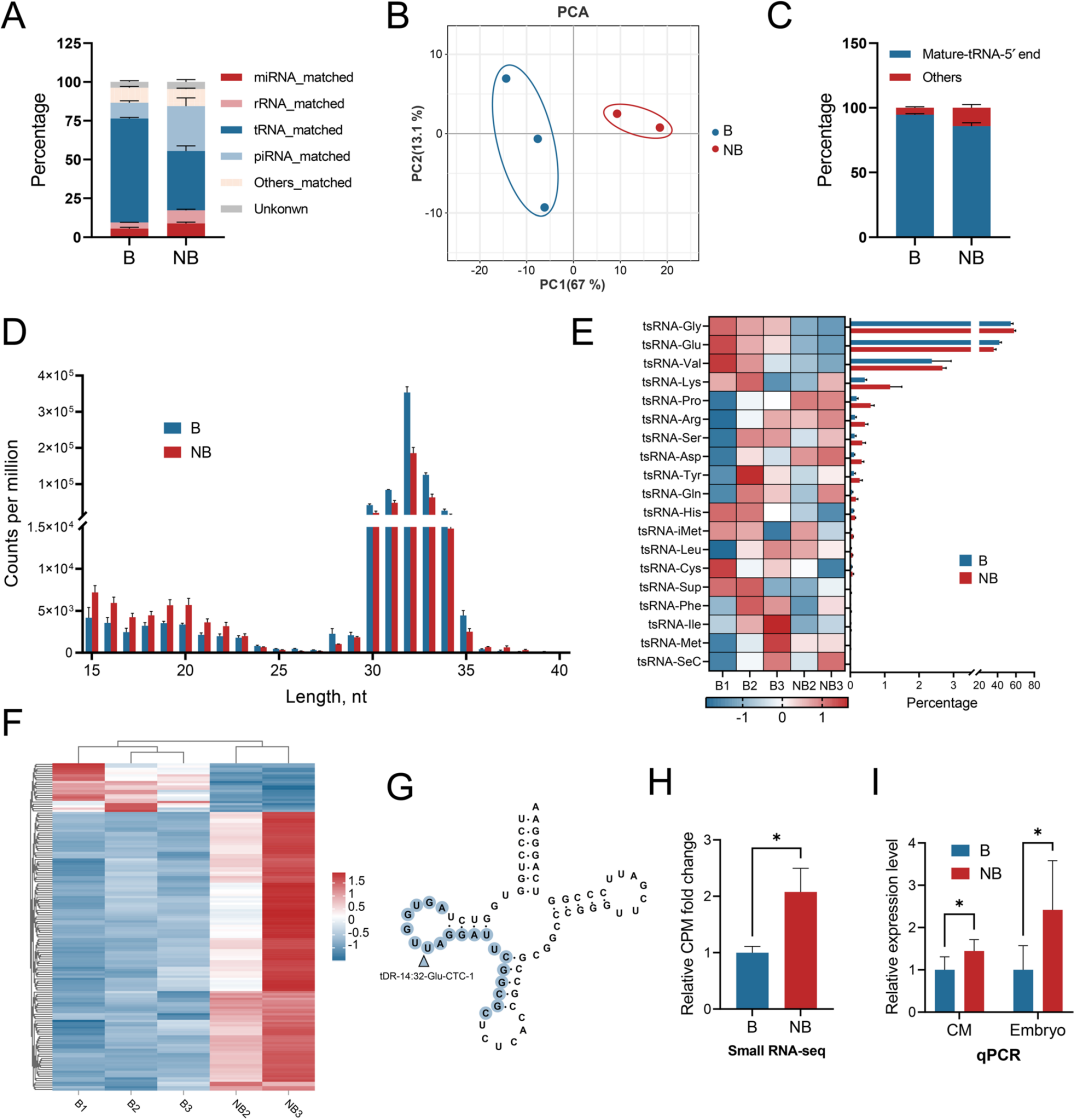
tsRNAs profiling of embryonic EVs derived from the conditioned medium of blastocysts (B) and non-blastocysts (NB). **A** sncRNAs mapping distribution of reads mapped to the *Bos taurus* genome (ARS-UCD1.2). **B** PCA of all 5 small RNA libraries based on tsRNAs expression. **C** Type percentages of tsRNAs. **D** Length distribution of reads mapped to tRNA. **E** Left: Heatmap showing the tsRNA relative expression levels (normalized to total miRNA levels and based on a Z-score transformed scale in the row direction) of five samples; Right: Percentage distribution of tsRNAs derived from the same tRNA. **F** Hierarchical clustering and heatmap of differentially expressed tsRNAs between B and NB. **G** A schematic diagram indicating the cleavage sites of tDR-14:32-Glu-CTC-1 from tRNA^Glu-CTC^. **H** Relative counts per million (CPM) fold change of tDR-14:32-Glu-CTC-1 in embryo derived EVs by small RNA-seq. **I** Relative expression level of tDR-14:32-Glu-CTC-1 in conditioned medium and embryos measured by RT-qPCR. Five biological replicates were used for RT-qPCR. Error bars represent SE. *P < 0.05

In order to investigate the tsRNA profile of EVs collected from the conditioned medium of embryos that reached the blastocyst (B) stage or not (non-blastocyst, NB), we revisited the small-RNA sequencing data from our previous study [5]. Notably, the distribution of sncRNAs revealed that miRNAs only represent a minor fraction of the total reads mapped to the genome, while tsRNAs are the dominant sncRNA type within the embryonic EVs (Fig. 1A). Principal component analysis (PCA) based on tsRNA expression unveiled a distinct profile discernible between the blastocyst and non-blastocyst group (Fig. 1B). The type distribution of tsRNAs showed that these molecules are primarily derived from mature tRNA 5´ ends (Fig. 1C). Additionally, a substantial expression increase was seen between 31 and 33 nucleotides (Fig. 1D), suggesting that 5´-tiRNAs rather than tRFs constitute the dominant tsRNA in EVs. Employing a heatmap representation, we further illustrated the relative amount of each tsRNA subcategory in the blastocyst and non-blastocyst group (Fig. 1E). The distribution pattern of the tsRNA subcategory showed that tRNA^Gly^ and tRNA^Glu^ are the main sources of tsRNAs in both groups (Fig. 1E). By comparing the tsRNA profiles, we identified 148 tsRNAs (126 up-regulated and 22 down-regulated) differentially expressed between EVs from blastocysts and non-blastocysts (Fig. 1F and Table S1). Among the differentially expressed tsRNAs, a 19 nt tRF derived from tRNA^Glu^ (Fig. 1G) showed a log_2_foldchange of 1.05 in the non-blastocyst EVs group (Fig. 1H). Subsequent RT-qPCR analysis confirmed this tRF, tDR-14:32-Glu-CTC-1, is significantly enriched in both conditioned medium and embryos of the non-blastocyst group (Fig. 1I). Collectively, these results suggest a potential role for tDR-14:32-Glu-CTC-1 in preimplantation embryo development.

**Fig. 2:**
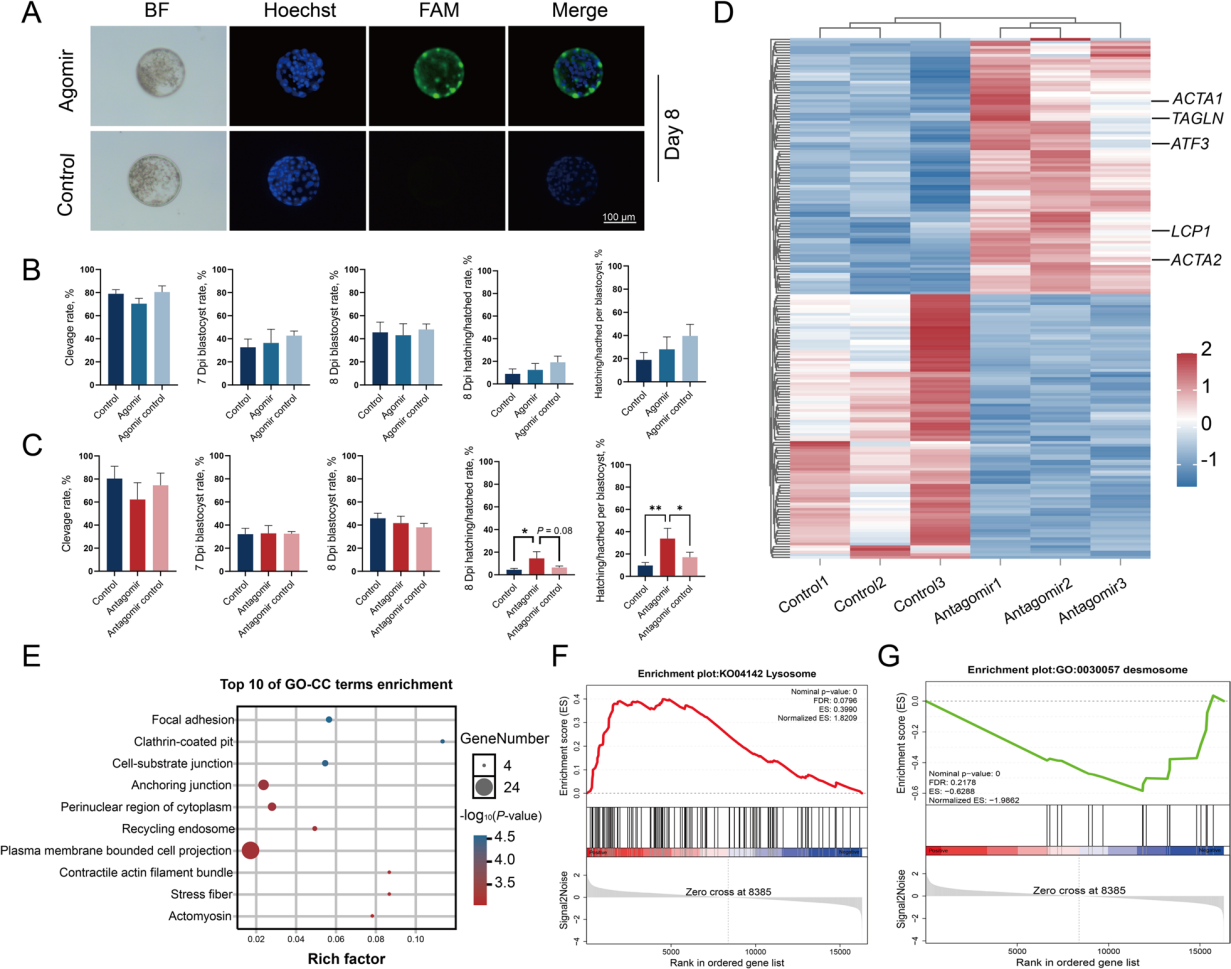
Inhibition of tDR-14:32-Glu-CTC-1 during preimplantation embryo development improves embryo hatching. **A** Fluorescence imaging showed tRF agomirs were taken up by embryos, BF: Bright-field, FAM: 5-Carboxyfluorescein. **B** Embryo developmental rates of bovine embryos treated with tDR-14:32-Glu-CTC-1 agomir. **C** Embryo developmental rates of bovine embryos treated with tDR-14:32-Glu-CTC-1 antagomir. **D** Hierarchical clustering and heatmap of DEGs between control group and tDR-14:32-Glu-CTC-1 antagomir group. **E** GO-Cellular Component terms enrichment of DEGs between control group and tDR-14:32-Glu-CTC-1 antagomir group. **F** Gene set enrichment analysis (GSEA) combined with the KEGG gene sets showed the lysosome pathway is up-regulated in tDR-14:32-Glu-CTC-1 antagomir group. **G** GSEA using the GO database showed the desmosome pathway is down-regulated in the antagomir group. Error bars represent SE. *P < 0.05, **P < 0.01

To validate the functional role of tDR-14:32-Glu-CTC-1 in preimplantation embryo development, we employed tRF agomirs or antagomirs, which are chemically modified tRF mimics or inhibitors, respectively. These molecules were supplemented to the in vitro conditioned medium containing presumed zygotes on 1 dpi and cultured until 8 dpi, thus allowing molecules to influence further embryo development. Fluorescence microscopy imaging showed that tRF agomirs were able to cross the zona pellucida, whereas the PBS control showed no signal (Fig. 2A). Notably, these labeled tRF agomirs were stable and detectable within the embryos up to 8 dpi. Furthermore, embryo development was not affected in the presence of tDR-14:32-Glu-CTC-1 agomir (Fig. 2B). Similarly, the supplementation of tDR-14:32-Glu-CTC-1 antagomir to the conditioned medium did not alter the cleavage or blastocyst rate either (Fig. 2C). However, inhibiting tDR-14:32-Glu-CTC-1 led to a significant increase in hatching rate compared to the control group (Fig. 2C). To gain deeper insight into the molecular mechanisms underlying the potential role of tDR-14:32-Glu-CTC-1 in embryo hatching, we performed transcriptome profiling comparing antagomir-treated blastocysts with the control group. RNA-seq revealed 195 genes were differentially expressed after the inhibition of tDR-14:32-Glu-CTC-1 (Fig. 2D and Table S2). Interestingly, inhibition of tDR-14:32-Glu-CTC-1 significantly increased the expression of genes related to actin cytoskeleton formation (*ACTA1*, *ACTA2*, *LCP1* and *TAGLN*) and embryo attachment (*ATF3*) (Fig. 2D). A comprehensive GO enrichment analysis showed the DEGs are significantly enriched within adhesion pathways (Fig. 2E). Additionally, GSEA combined with GO or KEGG gene sets showed the lysosome pathway was up-regulated while the desmosome pathway exhibited a down-regulation after the inhibition of tDR-14:32-Glu-CTC-1 (Fig. 2F–G).

## Discussion

tsRNAs exhibit a substantial presence in mature sperm as well as in human embryo-conditioned culture media, hinting at their potentially indispensable role during early embryogenesis [25, 26]. This study showed the tsRNA levels encapsulated within EVs derived from bovine blastocysts are more than 10-fold higher when compared to miRNAs. Previous studies have also noted the importance of tsRNAs in the early cleavage stages of mammalian embryos and down-regulation of tsRNAs may even cause embryonic lethality in zebrafish [13, 15]. However, these studies were all focused on tRNA halves instead of the shorter tsRNAs—tRFs. As a consequence, the roles of tsRNAs in preimplantation embryo development, particularly in hatching, remains largely unknown. Here, we studied a 19 nt tRF derived from tRNA^Glu^ by supplementing agomirs or antagomirs in the embryo conditioned medium and observed a significant increase in embryo hatching rate after tDR-14:32-Glu-CTC-1 inhibition.

Blastocyst hatching is an intricate process that can be regulated by the formation of actin-based trophectoderm projections [27]. In this study, the antagomir group exhibited an up-regulation of *ACTA1* and *ACTA2*, genes responsible for encoding actin proteins crucial in the formation of actin-based microfilaments that provide structural support for embryo hatching [28, 29]. Additionally, the knockdown of tDR-14:32-Glu-CTC-1 also led to increased expression of *LCP1* and *TAGLN*, genes encoding actin-binding proteins (lymphocyte cytosolic protein 1 and transgelin, respectively), thereby promoting actin assembly [30, 31]. In summary, the inhibition of tDR-14:32-Glu-CTC-1 resulted in heightened expression of actin-related genes, fostering the formation of actin-based trophectoderm projections and facilitating blastocyst hatching. Furthermore, activating transcription factor 3 (*ATF3)*, a transcription factor expressed in the human endometrium that can contribute to embryo attachment by transcriptionally increasing leukemia inhibitory factors expression [32], was significantly up-regulated after tDR-14:32-Glu-CTC-1 inhibition. Further substantiating our findings, these DEGs are enriched in pathways related to adhesion and the lysosome pathway, which are known facilitators of embryo implantation [33, 34]. Remarkably, the GSEA results in our study also highlighted the down-regulation of the desmosomes pathway in the antagomir group, aligning with earlier findings. Desmosomes are adhesive intercellular junctions that function in cell-to-cell adhesion. A decrease of desmosomal expression during the preimplantation stage was found previously in the uterine epithelium, leading to a diminished barrier function of the uterine epithelium against invading trophoblast cells. This subsequently facilitates the removal of the uterine epithelium and enables the developing embryo to penetrate the uterine stroma, thereby promoting successful implantation [35, 36]. Moreover, considering the potential impact of tsRNAs in embryo implantation and the communicative nature of EVs, it is conceivable that tRFs enclosed within EVs may extend their function beyond embryo development to maternal interactions that influence embryo reception.

## Conclusion

We revealed the abundance and potential regulatory ability of embryonic tsRNAs in EVs secreted by preimplantation embryos. We also found that the tRF molecule tDR-14:32-Glu-CTC-1 may play a pivotal role in regulating embryo development and implantation, as indicated by its effects on embryo hatching and its impact on the expression of genes associated with adhesion and embryo implantation-related pathways.

### Supplementary Information

**Additional file 1: Table S1.** Differentially expressed tsRNAs between blastocyst-EV group and non-blastocyst-EV group. **Table S2.** Differentially expressed genes between control group and antagomir group.

## Data Availability

The small-RNA sequencing data and transcriptomics data were deposited in the NCBI Gene Expression Omnibus database (accession ID: GSE197878) and Sequence Read Archive (accession ID: PRJNA1010691), respectively.

## Abbreviations

ACTA1: Actin alpha 1
ACTA2: Actin alpha 2
ATF3: Activating transcription factor 3
BSA: Bovine serum albumin
DEGs: Differentially expressed genes
dpi: Day post insemination
EVs: Extracellular vesicles
FAM: 5-Carboxyfluorescein
GO: Gene Ontology
GSEA: Gene set enrichment analysis
IETS: International embryo transfer society
ITS: Insulin, transferrin and selenium
IVF-TALP: In vitro fertilization—Tyrode’s albumin lactate pyruvate
KEGG: Kyoto Encyclopedia of Genes and Genomes
LCP1: Lymphocyte cytosolic protein 1
miRNA: MicroRNA
PBS: Phosphate buffered saline
PCA: Principal component analysis
PVP: Polyvinylpyrrolidone
sncRNAs: Small non-coding RNAs
SOF: Synthetic oviductal fluid
TAGLN: Transgelin
TCM-199: Tissue culture medium–199
tRF: tRNA fragment
tiRNAs: tRNA halves
tsRNAs: Transfer RNA-derived small RNAs
